# Supplementary material for: The Gut Bacteria Dysbiosis Contributes to Chronic Graft-Versus-Host Disease Associated With a Treg/Th1 Ratio Imbalance
Source: Front Microbiol. 2022 Sep 8;13:813576. doi: 10.3389/fmicb.2022.813576 (PMC9493085; doi:10.3389/fmicb.2022.813576)
Supplement: Supplementary file 1 [file Data_Sheet_1.zip › P101SC18090073-01-B1-3-4_result/05.WebShow/taxa_charts_group_html/charts/otmAfXx4C0dc54Zw5zrFCsWSlakkQ2_legend.pdf]

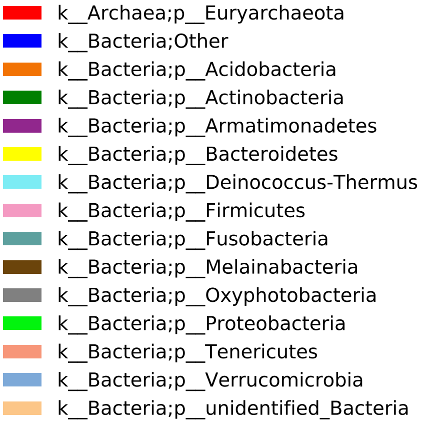
k__Archaea;p__Euryarchaeota
k__Bacteria;Other
k__Bacteria;p__Acidobacteria
k__Bacteria;p__Actinobacteria
k__Bacteria;p__Armatimonadetes
k__Bacteria;p__Bacteroidetes
k__Bacteria;p__Deinococcus-Thermus
k__Bacteria;p__Firmicutes
k__Bacteria;p__Fusobacteria
k__Bacteria;p__Melainabacteria
k__Bacteria;p__Oxyphotobacteria
k__Bacteria;p__Proteobacteria
k__Bacteria;p__Tenericutes
k__Bacteria;p__Verrucomicrobia
k__Bacteria;p__unidentified_Bacteria
